# Assessing the scalability of healthy eating interventions within the early childhood education and care setting: secondary analysis of a Cochrane systematic review

**DOI:** 10.1017/S1368980023002550

**Published:** 2023-12

**Authors:** Alice Grady, Jacklyn Jackson, Luke Wolfenden, Melanie Lum, Sze Lin Yoong

**Affiliations:** 1 School of Medicine and Public Health, University of Newcastle, Callaghan, Australia; 2 Population Health Research Program, Hunter Medical Research Institute, New Lambton, Australia; 3 Hunter New England Population Health, Hunter New England Local Health District, Wallsend, Australia; 4 National Centre of Implementation Science, University of Newcastle, Callaghan, Australia; 5 Global Centre for Preventive Health and Nutrition, Institute for Health Transformation, Deakin University, Victoria, Australia

**Keywords:** Scalability, Healthy eating, Early childhood education, Systematic review, scale-up

## Abstract

**Objective:**

Early childhood education and care (ECEC) is a recommended setting for the delivery of health eating interventions ‘at scale’ (i.e. to large numbers of childcare services) to improve child public health nutrition. Appraisal of the ‘scalability’ (suitability for delivery at scale) of interventions is recommended to guide public health decision-making. This study describes the extent to which factors required to assess scalability are reported among ECEC-based healthy eating interventions.

**Design:**

Studies from a recent Cochrane systematic review assessing the effectiveness of healthy eating interventions delivered in ECEC for improving child dietary intake were included. The reporting of factors of scalability was assessed against domains outlined within the Intervention Scalability Assessment Tool (ISAT). The tool recommends decision makers consider the problem, the intervention, strategic and political context, effectiveness, costs, fidelity and adaptation, reach and acceptability, delivery setting and workforce, implementation infrastructure and sustainability. Data were extracted by one reviewer and checked by a second reviewer.

**Setting:**

ECEC.

**Participants:**

Children 6 months to 6 years.

**Results:**

Of thirty-eight included studies, none reported all factors within the ISAT. All studies reported the problem, the intervention, effectiveness and the delivery workforce and setting. The lowest reported domains were intervention costs (13 % of studies) and sustainability (16 % of studies).

**Conclusions:**

Findings indicate there is a lack of reporting of some key factors of scalability for ECEC-based healthy eating interventions. Future studies should measure and report such factors to support policy and practice decision makers when selecting interventions to be scaled-up.

Dietary risk factors, including inadequate intakes of fruits, vegetables, whole grains and excessive intakes of unhealthy foods (foods high in added sugar, Na and saturated fat), are the leading contributors to death and disability globally^(1)^. Dietary intake in early childhood has implications for child physical, social and mental well-being^(2)^, placing children at an increased risk of developing a variety of non-communicable conditions later in life, including obesity and high blood pressure^(3,4)^. As the dietary behaviours and food preferences learnt during early childhood frequently carry through into adulthood^(5–7)^, improving the diet of young children is paramount to reduce the burden of dietary risk factors in the population.

Early childhood education and care (ECEC) settings (inclusive of long day cares, preschools, nurseries, kindergartens and family day care) provide access to a large number of young children (United States (US) ∼ 60 % of children^(8)^; Australia ∼50 % of children^(9)^), for prolonged and regular periods of time (on average 30 h per week), during a highly influential life stage^(10,11)^. As these settings are accessed by children and families across various socio-economic and demographic groups, they provide an opportunity to address health inequities in young children. Further, national regulations and quality assessment systems for the sector (e.g. National Quality Framework in Australia^(12)^, Quality Rating and Improvement System in the US^(13)^) support the creation of environments that promote healthy eating behaviours. As such, ECEC is recommended by the WHO as an important setting for the implementation of public health nutrition interventions^(14)^.

Over the past few decades, there has been considerable public health and research investment in the development and implementation of effective population-based interventions for improving child nutrition^(15–17)^. Despite evidence of efficacy of these interventions, assessments of their ‘real-world’ effectiveness demonstrate substantially reduced effects on child nutrition^(18)^. A recent systematic review assessing the effectiveness of scaled-up public health nutrition interventions found effect sizes reported from scaled-up interventions were on average only 50 % of the effect size reported in preceding efficacy trials^(18)^. Unless such interventions can be successfully scaled-up whilst maintaining an effect that is meaningful to the population, they offer little benefit and represent significant research waste. While a range of factors, such as poor reach, lack of intervention adherence, fidelity and dose, may contribute to the reduced effects of these scaled-up interventions, the limited impact may also be due, in part, to selection and subsequently implementation, of interventions that are not well suited to the contexts in which they are to be delivered for population scale-up. Such interventions are therefore likely to encounter a range of barriers to implementation at scale.

To provide evidence to support policy makers and practitioners to more readily assess whether ECEC-based healthy eating interventions are amenable for scale, assessment of intervention scalability is recommended^(19)^. Scalability is defined as ‘the ability of a health intervention shown to be efficacious on a small scale and or under controlled conditions to be expanded under real world conditions to reach a greater proportion of the eligible population, while retaining effectiveness’^(20)^. A range of tools have been designed to support scalability assessments^(21)^. Such tools suggest that in addition to intervention efficacy/effectiveness, other factors are thought to influence decision making regarding the scalability of public health interventions. These factors include the expertise and resources required to deliver the intervention outside of the research environment, potential reach, cost, availability of delivery infrastructure, as well as fit within the local context^(22,23)^.

The reporting of data relevant to the factors of scalability as part of trials of nutrition interventions in ECEC would better inform scalability assessments to support public health decision making. Such information is crucial for end-users to increase the likelihood of selecting an intervention that can be successfully scaled-up to produce public health impact^(24,25)^. However, the extent to which such information is available within published reports of healthy eating interventions in this setting is unknown. A number of previous reviews in the ECEC setting have extracted some information relevant to intervention scalability^(15,26,27)^; however, no previous reviews have sought to systematically examine the reporting of all scalability factors. As such, the aim of this study was to assess the extent to which the factors required to assess scalability are reported among healthy eating interventions conducted within the ECEC setting.

## Methods

We undertook secondary data analysis^(28)^ of included studies identified by the Cochrane systematic review conducted by Yoong et al.^(15)^, which aimed to assess the effectiveness of healthy eating interventions delivered in ECEC settings for improving child dietary intake in children aged 6 months to 6 years. The repurposing of data included within high-quality systematic reviews has been recommended as a way of reducing research waste, identification of research gaps and a way of addressing important public health policy and practice questions^(28)^.

Briefly, as per the inclusion criteria outlined by Yoong et al.^(15)^, this included the following:

- Randomised controlled trials (RCT), including cluster-RCT, stepped-wedge RCT, factorial RCT, multiple baseline RCT and randomised crossover trials;
- Interventions conducted within the ECEC setting that offer care for children 6 months to 6 years, which includes formal paid care such as preschools, nurseries, long day cares, kindergartens and family day care services;
- Interventions conducted with a range of participants, including (but not limited to) children attending the ECEC service; parents, guardians, or carers of children, and professionals responsible for the care provided to children attending an ECEC service (e.g. service directors, educators, volunteers, cooks or other employed staff) and
- Healthy eating interventions containing a nutrition component that aims to influence child diet.

The current study was limited only to those studies included in the Cochrane review that reported on any child dietary intake outcomes which included consumption of food groups/specific foods; consumption of beverage types/specific beverages; intake of macronutrients and specific dietary components; overall diet quality and specific diet quality components. Studies not reporting such an outcome (including those that only reported on anthropometric outcomes) were excluded given the focus of this review. Included studies could be at any stage of scale-up (i.e. efficacy, effectiveness, implementation or dissemination) as long as they reported child dietary intake outcomes.

### Identification of supporting evidence

As information regarding scalability factors may be reported in a range of publications beyond the primary trial outcome publication, we sought to comprehensively capture all peer-reviewed publications associated with an intervention to inform scalability assessments. This included forward and backward citation searches in Scopus of the included studies. The aim of this search was to identify any additional published data or information related to the included studies, reporting on, but not limited to, intervention development; effectiveness; implementation; dissemination; feasibility/acceptability; adaptations/fidelity; sustainability and cost-effectiveness outcomes.

### Data extraction

Characteristics of included studies were extracted by pairs of independent reviewers, using Microsoft Excel, as per Yoong et al.^(15)^. Data including first author, year, country, study design, delivery setting and participants and name and brief description of the intervention were extracted. Similar to previous reviews assessing the scale-up of nutrition, and obesity prevention interventions^(18,29)^ and based on proposed scale-up pathways for public health interventions^(30)^, included studies were categorised as efficacy (primarily aiming to evaluate the effect of an intervention in ideal, controlled settings), effectiveness (primarily aiming to evaluate the effect of an intervention in real-world settings), implementation (primarily aiming to evaluate strategies to increase the uptake or adoption of an evidence-based intervention within real world settings) or dissemination (primarily aiming to evaluate the distribution of an intervention within real-world settings).

### Scalability assessment

The extent to which data relating to the factors of scalability were reported by included studies were extracted according to the Intervention Scalability Assessment Tool (ISAT)^(22,23)^. Such an approach has been undertaken by two recent reviews^(24,25)^. The ISAT^(23)^ was developed to support policy-makers and practitioners to make systematic assessments of the suitability of health interventions for scale-up within high-income country health and community settings. Briefly, the ISAT tool consists of three parts. Part A: considers the context in which the intervention is being deliberated for scale-up and consists of five domains: (1) the problem; (2) the intervention; (3) strategic/political context; (4) evidence of effectiveness and (5) intervention costs and benefits. Part B: explores the potential implementation and scale-up requirements and consists of five domains: (1) fidelity and adaptation; (2) reach and acceptability; (3) delivery setting and workforce; (4) implementation infrastructure and (5) sustainability. Part C: provides a brief summary of the information gathered in Parts A and B. All sections of included studies were reviewed for relevant data, including the Introductions, Methods, Results, Discussion, Conclusions, Acknowledgements, Funding, Conflicts of interest and Appendices/Supplementary material.

Review authors identified data related to key scalability domains as described in Table 1.

**Table 1:** Scalability domains, description and examples of relevant data for each domain

Scalability domain	Description	Examples of relevant data
The problem	Has the problem, who it affects and its impact been described?	Reporting of the burden of disease of poor diet, overweight or obesity (locally or at a population level).
The intervention	Have intervention aims/objectives and key elements been described?	Reporting of study aims, and components of the intervention to any extent.
Strategic and political context	Has the funding source been disclosed? Has the strategic, political and/or environmental context in which the intervention is delivered been described?	Reporting of the study funding body, or lack of funding. Reporting the presence of intervention fit within curriculum, guidelines, or climate within the setting.
Evidence of effectiveness	Has intervention effectiveness for the target outcome been reported?	Reporting of outcomes relating to child dietary intake (per inclusion criteria). Studies may also report adverse outcomes of the intervention or the relative advantage of the current intervention over any existing healthy eating interventions, including current practices being employed (usual care).
Intervention costs	Have intervention delivery costs and/or cost–benefit analyses been reported?	Reporting of the total costs of the intervention, components of intervention delivery, results of formal economic evaluations.
Fidelity and adaptation	Have intervention adaptations or modifications been described? Has fidelity to the intervention been reported?	Reporting of any planned modifications made to pilot versions of the intervention and/or unplanned adaptations to the intervention or its delivery. Studies may also report on the potential impact of adaptations/modifications. Reporting of the extent to which the intervention and/or its components were delivered to, or implemented by, participants as intended. This may also include a description of how intervention fidelity would be monitored or maintained for scale-up.
Reach and acceptability	Has the potential reach of the intervention to the target population been reported? Has the acceptability of the intervention to relevant end-users/stakeholders been reported?	Reporting of the sample participating in the intervention (e.g. the number and representativeness of services, children, staff, families receiving the intervention) and/or its evaluation (e.g. study consent and attrition rates), relative to the wider target population. Data that may inform projections or estimations of potential reach. Reporting results of quantitative or qualitative assessments of intervention acceptability.
Delivery setting and workforce	Has the setting and organisation and/or workforce involved in intervention delivery been described?	Reporting on the individuals and organisations involved in training and/or delivering the intervention to end users. This may include the number or description of qualifications of individuals involved.
Implementation infrastructure	Have the required infrastructure or operational requirements for scale-up of the intervention been described?	Reporting of facilities/ classrooms, staffing/ training required for scale-up and/or plans for widespread delivery of the intervention. Studies may also report on the infrastructure barriers to widespread implementation.
Sustainability	Has the sustainability of the intervention been reported?	Reporting on the long-term outcomes of the study (≥12 months post intervention, demonstrating the extent to which intervention effects may be sustained. The sustainability of the required infrastructure (including funding, resources, processes, delivery workforce) may also be reported.

As we were interested in identifying whether such data were reported, we only systematically extracted data regarding availability and for each domain reported it as No: Data not reported; Partial: Data partially reported (i.e. one of the two items assessed for the domain was reported); Yes: Data fully reported. Only those domains assessing multiple factors within a single domain could be assessed as Partial (i.e. strategic and political context, fidelity and adaptation and reach and acceptability). Given the comprehensive nature of the ISAT domains, brief examples of the type and extent of data reported for each of the scalability domains have been described narratively. Scalability assessments were undertaken by one reviewer (AG) and checked by a second reviewer (JJ). In the case that one reviewer was an author on included studies (AG), the second (JJ) and third reviewer (ML), undertook and checked the scalability assessments, respectively. Discrepancies between reviewers were reconciled by consensus.

### Analysis and synthesis

Review findings were synthesised narratively with descriptive statistics (frequencies and percentages) used to report the number of ISAT domains assessed as ‘Yes: Data fully reported’ for each study and the number of studies assessed as ‘Yes: Data fully reported’ ‘No: Data not reported’ and ‘Partial: Data partially’ reported for each of the ISAT domains.

## Results

**Fig. 1:**
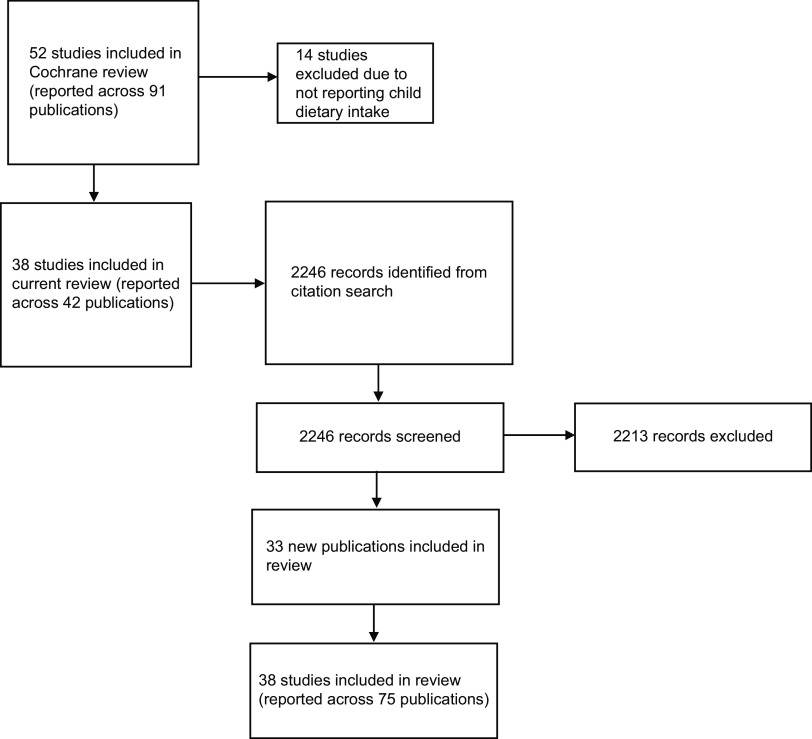
PRISMA diagram

A total of thirty-eight studies (reported across forty-two articles) were included from Yoong et al.; a subgroup of the total studies included in the Cochrane review^(15)^. Broadly, Yoong’s review found that healthy eating interventions in ECEC lead to small improvements in child diet quality and increased fruit consumption and vegetable consumption, however, did not have an effect on consumption of less healthy foods and sugar-sweetened drinks. A further 2246 titles were screened from the Scopus forward and backward citation search of included studies, identifying an additional thirty-three articles reporting relevant data, resulting in seventy-five included articles (see Fig. 1). Interventions were published between 2005 and 2022, and all were of a cluster RCT design. Interventions were most commonly conducted in the US (*n* 14), Australia (*n* 5), United Kingdom (*n* 2), Norway (*n* 2), Germany (*n* 2) and Belgium (*n* 2) (Table 2). All interventions were conducted within ECEC settings, fifteen of these included an additional component (beyond the intervention delivered in the ECEC setting) that was delivered in the child/family’s home^(31–45)^ and two included the wider community^(46,47)^. Thirty-three studies were categorised as effectiveness studies, with five categorised as implementation. None of the studies were categorised as efficacy or dissemination (Table 2). Brief descriptions of the characteristics of the interventions can be found in Table 2, along with additional articles associated with an intervention (identified via citation searching).

**Table 2:** Intervention characteristics and relevant associated publications

First Author, year, Intervention, Country	Design, Stage of scale-up	Delivery Setting. Population	Intervention description	Relevant associated publications
Baskale, 2011^(60)^, Not reported, Turkey	Cluster-RCT, Effectiveness	ECEC setting: 12 nursey schools; 238 children	6-week intervention involving game-based nutrition education for children.	N/A
Blomkvist, 2021^(55)^, Not reported, Norway	Cluster-RCT, Effectiveness	ECEC setting: 46 kindergartens; 267 children	Intervention 1: 3-month intervention involving repeated exposure of target vegetables. Services/kindergartens and parents had access to a website with recipes. Intervention 2: Intervention 1 plus, kindergarten staff were instructed to implement pedagogical tools including weekly sensory lessons with children.	Protocol: Blomkvist, 2018^(74)^
De Bock, 2012^(31)^, Not reported, Germany	Cluster-RCT, Effectiveness	ECEC + Home setting: 18 preschools; 377 children	6-month intervention involving nutrition sessions to children once a week. Five of the sessions involved parents, targeting them alone, and together with the children.	Protocol: De Bock, 2010^(93)^
DeCoen, 2012^(46)^, POP (Prevention of Overweight among Preschool and school children) Belgium	Cluster RCT, Effectiveness	ECEC + Wider Community: 6 communities; 31 preschools; 1589 children	2-year intervention focused on family, friends, pre-primary and primary schools, community stakeholders and local policy and media. Schools and teachers were provided resources to support the intervention program such as classroom activities; development of active playground; health related physical education; environmental and policy changes; providing education to parents, website, posters and letters.	NA
Fitzgibbon, 2005^(32)^, Hip-Hop to Health Jr. US	Cluster-RCT, Effectiveness	ECEC + Home setting: 12 Head Start sites; 409 children	14-week intervention including weekly healthy eating or exercise lessons and activities for children. Parents received newsletters and homework.	Protocol: Fitzgibbon, 2002^(71)^
Fitzgibbon, 2006^(33)^, Hip-Hop to Health Jr. US	Cluster-RCT, Effectiveness	ECEC + Home setting: 12 Head Start sites; 401 children	14-week intervention including weekly healthy eating or exercise lessons and activities for children. Parents received newsletters and homework. Intervention tailored to Latino population and delivered in both Spanish and English.	NA
Fitzgibbon, 2011^(34)^, Teacher-Based Hip-Hop to Health US	Cluster-RCT, Effectiveness	ECEC + Home setting: 18 preschools; 729 children	14-week intervention including teacher-delivered weekly healthy eating or exercise lessons and activities for children. Parents received newsletters and homework.	12 month follow-up: Kong, 2016^(94)^
Fitzgibbon, 2013^(35)^, Family-Based Hip-Hop to Health Jr. US	Cluster-RCT, Effectiveness	ECEC + Home setting: 4 preschools; 157 children	14-week intervention including weekly healthy eating or exercise lessons and activities for children + CD to supplement. Parents received newsletters, homework, CD + healthy eating and physical activity (PA) classes. Intervention adapted for lower-income, Hispanic populations.	NA
Gans, 2022^(56)^, Healthy Start-Comienzos Sanos US	Cluster-RCT, Effectiveness	ECEC setting: 119 FCCH; 377 children	8-month intervention in FCCH which included four components: 1. monthly support from a support coach trained in brief motivational interviewing; 2. tailored materials including a tailored report, newsletters and videos, in English or Spanish; 3. in-person group meetings every 6 weeks; and 4. a set of active toys.	Protocol: Risica, 2019^(95)^
Grummon, 2019^(36)^, Not reported, US	Cluster-RCT, Effectiveness	ECEC + Home setting: 4 centre-based childcare; 164 children	12-week intervention promoting consumption of healthier beverages with three main components: 1. environmental changes to classrooms; 2. implementation of rules and policies; 3. educational activities for children. Parents were invited to attend in-person training.	NA
Hu, 2010^(65)^, Not reported, China	Cluster-RCT, Effectiveness	ECEC setting: 7 kindergartens; 2102 children	12-month intervention that involved monthly nutrition education sessions; illustrated book distributed by teachers to children; nutrition and healthy lifestyle information provided to parents and promotional pictures displayed depicting common unhealthy and healthy dietary behaviours.	Exploratory analysis: Gao, 2014^(96)^
Iaia, 2017^(47)^, Not reported, Italy	Cluster-RCT, Effectiveness	ECEC + Wider Community: 16 childcare centres; 425 children	6-month intervention where teachers received tools and training to promote more healthy behaviours. Children and teachers engaged in learning experiences to 1. increase fruit and vegetable intake (e.g. repeated exposure; vegetable gardens; stories); 2. reduce time spent watching TV (e.g. book lending to stimulate reading at home); 3. limit sugar sweetened beverage intake (e.g. water was the only beverage at special events). Parents received information tools and motivational interviews with a trained paediatric nurse or primary care paediatrician.	NA
Jones, 2015^(61)^, Not reported, Australia	RCT, Implementation	ECEC setting: 128 childcare centres; 90 children	2-month intervention aimed at increasing childcare service implementation of 7 healthy eating and PA policies and practices, including implementation support, executive support, staff training, consensus processes, academic detailing visits, tools and resources, performance monitoring and feedback and a communications strategy.	Protocol: Jones, 2014^(97)^.
Kipping, 2019^(37)^, NAP SACC UK, UK	Cluster-RCT, Implementation	ECEC + Home setting: 12 nurseries; 177 children	5-month intervention based on the online Go NAP SACC adapted for the UK with childcare and at home components. ECEC components: 1. self-assessment completed by nursery manager; 2. workshop delivery to nursery staff on nutrition, oral health and PA; 3. action planning; 4. targeted technical assistance to help with goals; 5. review and reflection of action plans. Home components: Parents; 1. access to website; 2. assessment of food, drink, activity, oral health and sleep behaviours; 3. received tailored suggestions for goal setting; 4. received tailored information to help meet goals, and encouraged to review their goals.	Protocol: Kipping, 2016^(62)^. Process evaluation: Langford, 2019^(77)^.
Kobel, 2019^(38)^, Join the Healthy Boat, Germany	Cluster-RCT, Effectiveness	ECEC + Home setting: 57 kindergartens; 973 children	12-month intervention where kindergarten teachers received peer-to-peer training and resources including instructional and behavioural educational materials. Three key topics included the promotion of PA, reduction of screen time and healthy diet. Intervention materials included exercise and game lessons, ready to use ideas, action alternatives and lessons and family homework.	Protocol: Kobel, 2017^(98)^.
Kornilaki, 2021 ^(57)^, Not reported, Greece	Cluster-RCT, Effectiveness	ECEC setting: 15 nurseries; 329 children	4–6 week intervention developed to enhance young children’s knowledge of healthy eating, active play and environmental sustainability through play-based activities developed by nursery educators.	NA
Kristiansen, 2019^(39)^, BRA-study, Norway	Cluster-RCT, Effectiveness	ECEC + Home setting: 73 kindergartens; 633 children	5-month intervention to improve children’s vegetable intake at home and in kindergarten, including 1. training of kindergarten staff; 2. welcome package for kindergarten staff at training; 3. welcome package for the parent; 4. Website with materials for staff and parents; and 5. Facebook support group for parents and staff.	RCT-long-term effectiveness: Kristiansen, 2020^(99)^.
Leis, 2020^(51)^, Healthy Start Depart Sante (HSDS), Canada	Cluster-RCT, Effectiveness	ECEC setting: 61 licensed childcare centres or preschools; 897 children	6–8 month intervention, which included training and resources (i.e. implementation manual, PA, and healthy eating manuals and active play equipment kit), online/telephone support and monitoring for centres.	Protocol: Belanger, 2016^(100)^. RCT process evaluation: Ward, 2018^(75)^. Implementation evaluation: Ward, 2020^(101)^. Cost estimate: Sari, 2017^(102)^.
Lerner-Geva, 2015^(66)^, Not reported, Israel	Cluster-RCT, Effectiveness	ECEC setting: 6 kindergartens; 204 children	Intervention 1: 4-month intervention involving health eating and PA lessons. A summary of the lessons were provided to parents to reinforce the lessons. Teachers were trained to perform the lessons. Intervention 2: 10-week intervention involved lessons on healthy eating only. A summary of the lessons was provided to parents to reinforce the lessons. Teachers were trained to perform the lessons.	NA
Lumeng, 2017^(40)^, The Growing Healthy Study, US	Cluster-RCT, Effectiveness	ECEC + Home setting: 18 Head Start classrooms; 697 children	(1) HS (Head Start) (2) HS + POPS (Preschool Obesity Prevention Series) and (3) HS + POPS + IYS (Incredible Years Series). The POPS program targeted evidence-based obesity-prevention behaviours in the classroom (6 lessons over 12 weeks) and to parents, delivered by a trained nutrition educator, in collaboration with the classroom teacher. The IYS program targeted children’s self-regulation and was delivered in the classroom and to parents over approx. 7 months by a trained mental health specialist, in collaboration with teachers who participated in IYS teacher training.	Protocol: Miller, 2012^(103)^
Morris, 2018^(67)^, Not reported, Australia	Cluster-RCT, Effectiveness	ECEC setting: 25 teachers; 300 child–parent dyads	8-week intervention in which teachers implemented planned play-based healthy eating learning experiences.	NA
Namenek Brouwer, 2013^(69)^, Watch Me Grow, US	RCT, Effectiveness	ECEC setting: 4 centers; children not reported	4-month gardening intervention to promote vegetable and fruit intake among pre-schoolers.	NA
Natale, 2014^(52)^, Healthy Caregivers- Healthy Children (HC2) Phase 1, US	Cluster-RCT, Effectiveness	ECEC setting: 28 childcare centres; 1211 children	10-month intervention which consisted of environmental/centre modifications (i.e. menu and policy changes regarding drinks, snack, PA and screen time); a child curriculum (lesson plans); and a role modelling/gatekeeper curriculum for parents and teachers.	Protocol: Natale, 2013^(104)^ RCT long-term evaluation: Natale, 2017^(105)^ Phase 2 Protocol: Messiah, 2017^(72)^
Natale, 2021^(54)^, Healthy Caregivers-Healthy Children Phase 2, USA	Comparison of 2 RCT-cluster, Implementation	ECEC setting: 24 centres; 825 children	10-month intervention that consisted of environmental/centre modifications (i.e. menu and policy changes regarding drinks, snack, PA and screen time); a child curriculum (lesson plans) and a role modelling/gatekeeper curriculum for parents and teachers. Delivered via a train-the-trainer approach where university-based research team trained preschool-based coaches who in turn, trained childcare teachers to implement and disseminate the program.	Protocol: Messiah, 2017^(72)^
Nekitsing, 2019^(48)^, Not reported, UK	Cluster-RCT (2 × 2 factorial design), Effectiveness	ECEC setting: 11 preschools; 219 children	10-week intervention comparing the relative efficacy of repeated taste exposure, nutrition education, a combination of both conditions and no conditions (control), on intake of an unfamiliar vegetable in preschool-aged children.	NA
Pearson, 2022^(41)^ SWAP IT for Childcare, Australia	Cluster RCT, Effectiveness	ECEC + Home setting: 18 centre-based services; 400 children	10-week fully automated mobile communication intervention targeting parent’s packing of children’s lunchboxes in accordance with nutrition guidelines for the setting.	Protocol: Pond 2019^(106)^
Pinket, 2016^(59)^ The ToyBox-intervention, Europe (Belgium, Bulgaria, Germany, Greece, Poland, Spain)	Cluster RCT, Effectiveness	ECEC setting: 309 kindergartens; 4964 children	24-week intervention which examined the effects of the ToyBox-intervention: Teacher training sessions; ToyBox-intervention materials (e.g. classroom activity guide, hand puppets to implement fun classroom activities). The classroom activity guide consisted of three sections: setting environmental changes, pre-schoolers’ implementing the actual behaviour and teachers implementing fun classroom activities. Resources were provided to children to take home.	Protocol: Manios 2012^(107)^ Cochrane Review RCT: De Craemer 2020^(76)^
Puder, 2011^(42)^ Ballabeina, Switzerland	Cluster RCT, Effectiveness	ECEC + Home setting: 40 preschool classes; 727 children	1 year intervention targeting four lifestyle behaviours: PA, nutrition, media use and sleep. Trained health promoters intervened on the level of the teachers (workshops, visits with hands on training, assistance in the adaptation of the built environment), parents (events in collaboration with the teachers) and children (nutrition and PA lessons).	Protocol: Niederer 2009^(108)^
Ray, 2020^(63)^ DAGIS, Finland	Cluster RCT, Effectiveness	ECEC setting: 32 preschools; 802 children	23-week intervention involving resources and activities run in both preschools and homes and divided into five themes: Self-regulation (SR) skills; PA; fruit and vegetables; screen time and sugary foods and beverages.	Protocol: Ray 2019^(109)^
Reyes-Morales, 2016^(43)^ Not reported, Mexico	Cluster RCT, Effectiveness	ECEC + Home setting: 16 centres; 674 children	12-month intervention delivered using 3 components: training of childcare staff; educational sessions for children; workshops for parents.	NA
Roberts-Gray, 2018^(44)^ LunchBag, US	Cluster RCT, Effectiveness	ECEC + Home setting: 30 centres; 633 parent–child dyads	5-week + 1 booster (23 weeks later) multi-component, multi-level intervention to increase vegetables, fruit and wholegrains in preschool children’s lunches where parents supply a bag lunch.	Efficacy study: Roberts-Gray 2016^(110)^
Seward, 2018^(50)^ Not reported, Australia	Cluster RCT, Implementation	ECEC setting: 54 services; 395 children	6-month intervention to improve childcare service compliance with nutrition guidelines by addressing barriers and enablers to implementation.	Protocol: Seward, 2016^(111)^ Cochrane Review RCT: Yoong 2019^(112)^ 12 month follow-up: Grady, 2020^(73)^
Vaughn, 2020^(58)^ Healthy Me, Healthy We, US	Cluster RCT, Effectiveness	ECEC setting: 92 childcare centres; 853 children	8-month, social marketing intervention to encourage ECEC providers and parents to use practices that supported children’s healthy eating and PA behaviours.	Process evaluation: Luecking, 2021^(113)^ Protocol: Hennink-Kaminski, 2018^(114)^ Intervention development: Vaughn 2019^(115)^
Vereecken, 2009^(45)^ Beastly Healthy at School, Belgium	Cluster RCT, Effectiveness	ECEC + Home setting: 16 preschools; 1432 children	6-month healthy eating intervention program targeting (i) the class; (ii) schools via teachers and via the environment and (iii) the home environment.	NA
Ward, 2020^(64)^ Keys to Healthy Family Child Care Homes, US	Cluster RCT, Effectiveness	ECEC setting: 166 FCCH; 496 children	9-month intervention delivered to FCCHs using three modules addressing (1) FCCH provider health, (2) the FCCH environment (encouraged sharing education materials with families to help parents adopt similar changes at home) and (3) FCCH business practices (targeted finances).	Protocol: Ostbye et al. 2015^(116)^ Recruitment processes: Ward 2016^(117)^ Intervention development: Mann 2015^(118)^
Witt (2012)^(70)^ Color Me Healthy, US	Cluster RCT, Effectiveness	ECEC setting: 17 childcare centres; 263 children	6-week intervention of fun, interactive learning opportunities on PA and healthy eating using colour, music and exploration of the senses (including taste) to teach children, emphasising fruits and vegetables of different colours. Families received take home newsletters and activities.	NA
Yoong, 2020^(53)^ FeedAustralia, Australia	Cluster RCT, Implementation	ECEC setting: 35 centres; 522 children	12-month web-based menu planning intervention that supported childcare centre cooks align the provision of foods with dietary guidelines.	Protocol: Yoong 2017^(119)^ RCT non-diet outcome and process evaluation: Grady 2020^(120)^ Economic Evaluation: Reeves 2021^(121)^
Zeinstra, 2018^(68)^ Not reported, The Netherlands	Cluster RCT, Effectiveness	ECEC setting: 4 centres; 250 children	5-month intervention using repeated vegetable exposure to children to increase vegetable acceptance.	NA

NA, not applicable; RCT, randomised controlled trial; FCCH, Family Child Care Homes; PA, physical activity; ECEC, early childhood education and care; US, United States; UK, United Kingdom.

### Scalability of healthy eating interventions

None of the studies reported on all ten domains of scalability (Table 3). Across studies, the reporting of domains ranged between four to nine domains. In total, twenty-three (61 %) studies reported on more than half (i.e. > 5) of the domains of scalability.

**Table 3:** Scalability assessments of included studies according to ISAT domains

First Author, Year	The Problem	The Program/Intervention	Strategic/Political Context	Evidence of Effectiveness	Intervention Costs	Fidelity and Adaptation	Reach and Acceptability	Delivery Setting and Workforce	Implementation Infrastructure	Sustainability	Total number of domains reported	
											*n*	%
Başkale, 2011^(60)^	Yes^(60)^	Yes^(60)^	Yes^(60)^	Yes^(60)^	No	No	Partial Reach reported*^(60)^	Yes^(60)^	No	No	5	50
Blomkvist, 2021^(55)^	Yes^(55,74)^	Yes^(55,74)^	Partial Funding sources reported*^(55)^	Yes^(55)^	No	Yes^(55)^	Partial Reach reported*^(55)^	Yes^(55)^	Yes^(55)^	No	6	60
De Bock, 2012^(31)^	Yes^(31)^	Yes^(31)^	Yes^(31,93)^	Yes^(31)^	No	Yes^(31)^	Partial Reach reported*^(31)^	Yes^(31)^	No	No Sustainability data being measured^(31)^, but not yet reported*	6	60
De Coen, 2012^(46)^	Yes^(46)^	Yes^(46)^	Partial Funding sources reported*^(46)^	Yes^(46)^	No	Partial Fidelity briefly reported*^(46)^	Partial Reach reported*^(46)^	Yes^(46)^	No	No	4	40
Fitzgibbon, 2005^(32)^	Yes^(32,71)^	Yes^(32,71)^	Partial Funding sources reported*^(32,33,71)^	Yes^(32,33)^	No	Yes^(32,71)^	Partial Reach reported*^(33)^	Yes^(32,71)^	No	Yes^(32)^	6	60
Fitzgibbon, 2006^(33)^	Yes^(33)^	Yes^(33)^	Partial Funding sources reported*^(33)^	Yes^(33)^	No	Partial Fidelity reported*^(33)^	Partial Reach reported*^(33)^	Yes^(33)^	No	Yes^(33)^	6	60
Fitzgibbon, 2011^(34)^ & Kong, 2016^(94)^	Yes^(34,94)^	Yes^(34,94)^	Partial Funding sources reported*^(34,94)^	Yes^(34,94)^	No	Yes^(34)^	Yes^(33,34,94)^	Yes^(34)^	Yes^(34,94)^	No	7	70
Fitzgibbon, 2013^(35)^	Yes^(35)^	Yes^(35)^	Yes^(35)^	Yes^(35)^	No	Partial Adaptations reported*^(35)^	Partial Reach reported*^(35)^	Yes^(35)^	Yes^(35)^	No	6	60
Gans, 2022^(56)^	Yes^(56,95)^	Yes^(56,95)^	Yes^(56,95)^	Yes^(56)^	No	Partial Fidelity reported*^(56)^	Yes^(56)^	Yes^(56,95)^	Yes^(95)^	No	7	70
Grummon, 2019^(36)^	Yes^(36)^	Yes^(36)^	Yes^(36)^	Yes^(36)^	No	Partial Fidelity reported*^(36)^	Partial Reach reported*^(36)^	Yes^(36)^	No	No	5	50
Hu, 2010^(65)^	Yes^(65)^	Yes^(65)^	Partial Funding source reported*^(65)^	Yes^(65)^	No	No	Yes	Yes^(65,96)^	No	No	5	50
Iaia, 2017^(47)^	Yes^(47)^	Yes^(47)^	Partial Funding source reported*^(47)^	Yes^(47)^	Yes^(47)^	No	Yes^(47)^	Yes^(47)^	Yes^(47)^	Yes^(47)^	8	80
Jones, 2015^(61)^	Yes^(61,97)^	Yes^(61,97)^	Yes^(61,97)^	Yes^(61)^	No	Partial Fidelity reported*^(61)^	Yes^(61)^	Yes^(61)^	No	No	6	60
Kipping, 2019^(37)^	Yes^(37,62,77)^	Yes^(37,62,77)^	Yes^(37,62,77)^	Yes^(37)^	Yes^(37,77)^	Yes^(37,77)^	Yes^(37,62,77)^	Yes^(77)^	Yes^(37,62,77)^	No	9	90
Kobel, 2019^(38)^	Yes^(38,98)^	Yes^(38,98)^	Partial Funding source reported*^(38,98)^	Yes^(38)^	No	No	Partial Reach reported*^(38,98)^	Yes^(38,98)^	Yes^(38)^	No	5	50
Kornilaki, 2021^(57)^	Yes^(57)^	Yes^(57)^	No	Yes^(57)^	No	No	Partial Reach reported*^(57)^	Yes^(57)^	Yes^(57)^	No	5	50
Kristiansen, 2019^(39)^ & 2020^(99)^	Yes^(39)^	Yes^(39)^	Partial Funding source reported*^(39)^	Yes^(39,99)^	No	No	Partial Reach reported*^(39)^	Yes^(39)^	Yes^(39)^	Yes^(99)^	6	60
Leis, 2020^(51)^	Yes^(51,75,100)^	Yes^(51,75,100)^	Yes^(51,75,100)^	Yes^(51)^	Yes^(101,102)^	Yes^(75)^	Yes^(51,75,100)^	Yes^(51)^	Yes^(51)^	No	9	90
Lerner Geva, 2015^(66)^	Yes^(66)^	Yes^(66)^	Partial Funding source reported*^(66)^	Yes^(66)^	No	No	No	Yes^(66)^	Yes^(66)^	No	5	50
Lumeng, 2017^(40)^	Yes^(40,103)^	Yes^(40)^	Yes^(40,103)^	Yes^(40)^	No	Partial Fidelity reported*^(40)^	Yes^(40)^	Yes^(40)^	Yes^(103)^	No	7	70
Morris, 2018^(67)^	Yes^(67)^	Yes^(67)^	Partial Political/ strategic context reported*^(67)^	Yes^(67)^	No	No	No	Yes^(67)^	No	No	4	40
Namenek Brouwer, 2013^(69)^	Yes^(69)^	Yes^(69)^	No	Yes^(69)^	No	No	Partial Acceptability reported*^(69)^	Yes^(69)^	No	No	4	40
Natale, 2014^(52)^	Yes^(52,104,105)^	Yes^(52,104,105)^	Partial Funding sources reported*^(52,104,105)^	Yes^(52)^	Yes^(72)^	No Fidelity data being collected*^(104,105)^	Partial Reach reported. Acceptability data being collected*^(72,105)^	Yes^(54,104)^	No	Yes^(105)^	6	60
Natale, 2021^(54)^	Yes^(54,72)^	Yes^(54,72)^	Yes^(54,72)^	Yes^(54)^	No	Yes^(54,72)^	Partial Reach reported*^(54,72)^	Yes^(54)^	Yes^(54,72)^	Yes^(54)^	8	80
Nekitsing, 2019^(48)^	Yes^(48)^	Yes^(48)^	Partial Funding source reported*^(48)^	Yes^(48)^	No	Partial Fidelity reported*^(48)^	Yes^(48)^	Yes^(48)^	No	No	5	50
Pearson, 2022^(41)^	Yes^(41,106)^	Yes^(41,106)^	Yes^(41,106)^	Yes^(41)^	No Cost-effectiveness analysis planned, but not conducted*^(41)^	Yes^(41)^	Yes^(41)^	Yes^(41,106)^	Yes^(106)^	No	8	80
Pinket, 2016^(59)^ & De Craemer, 2020^(76)^	Yes^(59,76)^	Yes^(59,76,107)^	Yes^(59,76,107)^	Yes^(59,76)^	No Health economic modelling being used to assess cost-effectiveness^(107)^ but not yet reported*	Partial Need for local/cultural adaptations noted*^(76)^ Fidelity reported*^(59,76)^	Yes^(59,76,107)^	Yes^(59,76)^	No	No	6	60
Pruder, 2011^(42)^	Yes^(42,108)^	Yes^(42)^	Partial Funding source reported*^(42,108)^	Yes^(42)^	No	Yes^(42)^	Yes^(42)^	Yes^(42)^	No	No	6	60
Ray, 2020^(63)^	Yes^(63,109)^	Yes^(63,109)^	Yes^(63,109)^	Yes^(63)^	No	No Fidelity will be measured^(109)^, but not yet reported*	Partial Reach reported*^(63)^ Acceptability will be measured^(109)^, but not yet reported	Yes^(63)^	No	No	5	50
Reyes-Morales, 2016^(43)^	Yes^(43)^	Yes^(43)^	Partial Funding source reported*^(43)^	Yes^(43)^	No	No	No	Yes^(43)^	No	No	4	40
Roberts-Gray, 2018^(44)^	Yes^(110)^	Yes^(110)^	Partial Funding source reported*^(44,110)^	Yes^(44)^	No	Partial Fidelity reported*^(44,110)^	Yes^(44,110)^	Yes^(110)^	No	No	5	50
Seward, 2018^(50)^, & Yoong 2019^(112)^	Yes^(50,111,112)^	Yes^(50,111,112)^	Yes^(50,111,112)^	Yes^(50,112)^	No ^(112)^	Partial Fidelity reported*^(50)^	Partial Reach reported*^(50,112)^	Yes^(50,111)^	Yes^(112)^	No^(73)^	6	60
Vaughn, 2020^(58)^	Yes^(58,113–115)^	Yes^(58,114,115)^	Partial Funding source reported*^(58,114)^	Yes^(58)^	No Only very brief estimate of some resource costs provided*^(114)^	Yes^(58,113)^	Partial Acceptability reported*^(58,113)^	Yes^(58)^	Yes^(113)^	No	6	60
Vereecken, 2009^(45)^	Yes^(45)^	Yes^(45)^	Yes^(45)^	Yes^(45)^	No	No	Yes^(45)^	Yes^(45)^	No	No	6	60
Ward, 2020^(64)^	Yes^(64)^	Yes^(64)^	Yes^(64,117,118)^	Yes^(64)^	No	Partial Fidelity reported*^(64)^	Yes^(64,117)^	Yes^(64,116)^	No	No	7	70
Witt, 2012^(70)^	Yes^(70)^	Yes^(70)^	No	Yes^(70)^	No	Partial Fidelity reported*^(70)^	Partial Acceptability reported*^(70)^	Yes^(70)^	No	No	4	40
Yoong, 2020^(53)^	Yes^(53,119–121)^	Yes^(53,119,120)^	Yes^(53,119,120)^	Yes^(53)^	Yes^(121)^	Yes^(120)^	Yes^(53,120)^	Yes^(53)^	Yes^(53,119,120)^	No	9	90
Zeinstra, 2018^(68)^	Yes^(68)^	Yes^(68)^	Partial Funding source reported*^(68)^	Yes^(68)^	No	Partial Fidelity reported*^(68)^	No	Yes^(68)^	No	No	4	40
Total number of studies fully reporting domain												
* n*	38	39	17	38	5	13	16	38	17	6		
%	100	100	45	100	13	34	42	100	45	16		

All thirty-eight (100 %) studies described the problem, the intervention objective and key elements, the effectiveness of the intervention and the delivery workforce and setting. For all studies, ‘the problem’ was reported as the burden of disease and prevalence of poor dietary intake for the population of interest. While the intervention objectives were clearly described for all studies, the amount of detail describing the intervention elements was variable. For example, some studies provided only a brief description of the intervention^(48)^ whereas others provided detailed accounts of each intervention component^(39,41)^ and reported according to TIDieR (template for intervention description and replication) guidelines^(49)^. All studies reported on intervention effectiveness in improving child dietary intake (per inclusion criteria); however, few reported whether the intervention resulted in any adverse outcomes (e.g. negative impacts on child health or staff/parent attitudes)^(32,33,37,41,50)^. None described the relative advantage of the intervention being evaluated over existing interventions to address child dietary intake in the setting (e.g. comparison of any perceived differences in the strategic, political, economic or societal outcomes of the intervention over usual practice and/or alternate healthy eating interventions delivered in ECEC). The extent of information reported also varied for the delivery workforce and setting. While all studies reported the setting (ECEC, home and wider community) and the workforce delivering the intervention (most commonly ECEC staff, researchers and external nutrition experts), reporting of the number and description of formal qualifications of the individuals involved in delivering the interventions was variable^(42,46)^.

Only five (13 %) studies reported on the cost domain. This reporting included the cost of delivering the intervention^(37,47,51)^ and formal cost analyses (i.e. cost-effectiveness and cost-utility)^(52,53)^. Regarding the sustainability domain, only six (16 %) studies reported on the sustainability of the intervention (i.e. assessed as reporting study outcomes ≥ 12 months post intervention^(32,33,39,47,52,54)^. The sustainability of the required infrastructure (including funding, resources, processes and delivery workforce) for intervention delivery, however, was rarely reported.

Implementation infrastructure was reported for seventeen (45 %) of the studies^(34,35,37–41,47,48,51,53–58)^. The extent of information and content of this domain varied substantially. For example, some studies reported the intervention was already being scaled^(38,42,59)^, albeit little detail on the infrastructure and operational requirements for scale-up were provided, whereas others reported on the resource barriers to widespread implementation of the intervention^(37,55)^.

Three ISAT domains (political and strategic context, fidelity and adaptation and reach and acceptability) contained two criteria, and therefore could receive a ‘Partial’ rating. Seventeen (45 %) studies fully reported on the political and strategic context domain of the ISAT^(31,35,36,40,41,45,50,51,53,54,56,59–64)^, eighteen (47 %) studies reported partial data^(32–34,38,39,42–44,46–48,52,55,58,65–68)^ and three (8 %) studies did not report this domain at all^(57,69,70)^. For those studies partially reporting data, this included one study only reporting context and seventeen studies only reporting on funding sources. Overall, studies reporting on the context surrounding the intervention (*n* 18) provided varying accounts (e.g. alignment of the intervention into mandatory nutrition curriculum^(60)^ policies and guidelines for the ECEC setting^(53)^, inclusion in state-sponsored nutrition programmes^(31)^, support from local, state and national governing organisations^(45,64)^. The reporting of the source of funding received (or lack thereof) (*n* 34) was fairly consistent across studies.

Fidelity and adaptation were reported in full by 34 % of studies (*n* 13)^(31,32,34,37,41,42,51,53–55,58)^, partially reported by 32 % of (*n* 12) studies^(33,35,36,40,44,46,48,50,56,59,61,64,68,70)^ and not at all by 34 % (*n* 13) of studies^(38,39,43,45,47,52,57,60,63,65–67,69)^. For those studies partially reporting data, this included eleven studies only reporting fidelity, and one study only reporting adaptations. Overall, the studies that reported on intervention fidelity (*n* 24), most often described compliance in delivery of the intervention components from the delivery workforce^(31,61)^, or implementation of the intervention among intervention recipients (i.e. staff and parents)^(40,70)^. None of the studies reported how intervention fidelity would be monitored or maintained long term (e.g. any existing structures/processes or future plans for the monitoring or maintenance of intervention delivery). Overall, the reporting of adaptations of the interventions (*n* 13) covered planned modifications from pilot interventions^(55,71,72)^, in addition to unplanned adaptations during the intervention period^(42,73)^. The likely impact of these unplanned modifications on intervention effectiveness was rarely described^(42)^.

Reach and acceptability were reported in full for sixteen (42 %) studies^(34,37,40–42,44,45,47,48,51,53,56,59,61,64,65)^, partially for eighteen (47 %) studies^(31–33,35,36,38,39,46,50,52,54,55,57,58,60,63,69,70)^ and was not reported for four (11 %) studies^(43,66–68)^. For those studies partially reporting data, this included fifteen studies only reporting reach and three studies only reporting acceptability. Overall, of those studies reporting reach (i.e. the number and representativeness of participants, relative to the target population) (*n* 31), this was often reported in the context of the trial evaluation (e.g. consent and attrition rates)^(63,74)^, rather than reach of the intervention to ECEC services, staff, children and parents (if applicable)^(75)^. Overall, reporting on the acceptability (*n* 19) of the intervention (or components of) among any end-user or stakeholder was most commonly from the perspective of ECEC staff and parents. Intervention acceptability included formal measurement via questionnaires^(59,76)^ or qualitative interviews^(37,77)^ with participants with findings reported in study results and brief statements of acceptability reported in discussions^(47,65)^.

## Discussion

This is the first study to assess the extent to which the factors required to assess scalability have been reported among healthy eating interventions in the ECEC setting. We found that despite a substantial number of RCTs evaluating the impact of healthy eating interventions on child dietary intake, the reporting of factors important to assess scalability within these interventions is scarce, with no studies reporting on all ten factors assessed. Across studies, the reporting of domains ranged between four and nine domains. In total, twenty-three (61 %) studies reported on more than half (i.e. > 5) of the domains of scalability. The studies reporting the highest number of scalability factors were Yoong et al.’s feedAustralia^(53)^, Leis et al.’s Healthy Start Départ Santé^(51)^ and Kipping et al.’s NAPSACC UK^(37)^. These three studies fully reported on all factors, with the exception of sustainability, and were published between 2019 and 2020 – more recently than other studies included in this review. Further two of these studies were classified to be at the ‘implementation’ stage of scale-up. These findings may be a result of the growing prominence of implementation research, guidance on developing implementation strategies^(78)^ and measuring implementation outcomes in this setting^(21)^, in addition to the benefits of employing hybrid designs to simultaneously evaluate intervention effectiveness and implementation^(79)^, which are aligned to some of the factors recommended to assess intervention scalability. This finding also highlights that the opportunity and appropriateness of reporting domains of scalability may differ based on the type of study and stage of scale-up. As trials move through the translational pipeline from efficacy through to dissemination the focus becomes less about the internal validity of an intervention, with greater emphasis on external validity, and therefore broad assessments of intervention impact in the real world (with greater consideration to scalability domains such as acceptability, reach for example).

In terms of individual factors to assess scalability, we found the problem, the intervention, effectiveness and the delivery workforce and setting were the most frequently reported, with relatively low reporting of the domains of fidelity and adaptation and reach and acceptability among included studies. These findings are broadly similar to reviews assessing the scalability of home telemonitoring-based interventions^(25)^ and infant obesity prevention interventions^(24)^, also based on the ISAT. Data relating to the cost and sustainability domains, however, were the least reported factors, with only 13 % and 16 % of included studies reporting these, respectively. These findings are similar to reviews assessing implementation interventions within the ECEC setting^(26,27)^, however, are in contrast to reviews outside of the ECEC setting which found the cost domain to be reported in 43–77 % of studies and sustainability reported in 50–77 % of studies^(24,25,80)^. The practical implications of these findings are substantial as this lack of information means that decisions whether to scale-up ECEC-based healthy eating interventions (or not) are being made in the absence of critical evidence regarding budgets and infrastructure (resources, including processes and delivery workforce) required to implement these interventions and the longer-term impact (or lack thereof) of such interventions. Consideration of the long-term availability of the required infrastructure for intervention delivery, alongside the use of guides and frameworks to support the development and selection of implementation strategies likely to facilitate intervention sustainability, may represent examples of how researchers can plan for sustainability^(78)^.

It is important to recognise that the variability in reporting of scalability factors within the current, and across other reviews, may be due to the type of information conventionally reported within journal articles, with some domains (particularly those related to implementation) only receiving more attention in recent years. There are also substantial challenges for researchers in terms of being able to measure and report on every factor of scalability while considering participant burden and funding constraints. Often the limited and competitive funding for research is insufficient to cover the costs for collection of data relating to all domains of scalability, in particular long-term follow-up (sustainability) or for formal economic evaluations. Further, design requirements of included studies (e.g. presence of a control arm) and challenges in conducting comparative effectiveness and factorial trials likely contribute to the lack of reporting regarding the relative advantage of the intervention over existing interventions (within the effectiveness domain). Previous research suggests there are differing levels of perceived importance of scalability domains across different health conditions, settings, contexts and individuals (researchers, policy-makers and practitioners)^(20)^. While differing levels of importance have been identified for public health^(81)^ and nutrition and physical activity interventions broadly^(82)^, this is yet to be explored within the ECEC setting specifically.

As the weighting of scalability domains is likely to impact recommendations on whether an intervention should be scaled-up or not^(25,83)^, investigation into the relative importance of some factors of scalability to decision makers and how these should be defined and measured^(21,25)^, in the ECEC setting is warranted. This should be conducted from multiple perspectives, including researchers, policy makers, practitioners, funding bodies, ECEC staff, ECEC governing and advocacy bodies (e.g. the Australian Children’s Education and Care Quality Authority, Canada’s Federal Secretariat on Early Learning and Child Care, the Child and Adult Care Food Program in the US), and the community^(21)^. Such information could guide future reporting of public health nutrition interventions.

Given the failings of public health nutrition interventions to retain their effectiveness when implemented at scale^(18,84)^, it is recommended that interventions be designed, evaluated and reported with scalability in mind^(85)^. The process of designing for scale and evaluating scalability, in addition to the outcomes of intervention scalability assessments, should be reported and published^(80)^ to facilitate transparency and support decision making by policy makers and practitioners looking to implement and scale public health interventions^(83,86,87)^. A recent brief by Barnes et al.^(86)^ provides an example of this, detailing how scalability was prioritised within the evaluation of a web-based program to improve child nutrition in ECEC, with a scalability assessment guided by the ISAT. A number of other avenues may also facilitate improvements in the reporting of factors of scalability. For example, policy and practice decision-makers should advocate and/or require such processes and data be reported or collected, prior to selecting public health interventions to be scaled-up. In addition, funding bodies and journals could employ guidelines which prioritise the evaluation and reporting of such data. For example, the SUCCEED project (standards for reporting studies assessing the impact of scaling strategies) aims to develop reporting guidelines for scaling studies and could be recommended for studies that have a public health application^(88)^.

### Strengths and limitations

A number of limitations in the design of the current study need to be acknowledged. First, included studies were restricted to those identified in a previous review. Some relevant studies reporting on healthy eating interventions in ECEC (not meeting the Cochrane systematic review criteria) may therefore not be captured here; however, it is likely this review provides a comprehensive list of all ECEC-based healthy eating RCTs. Second, the appropriateness of assessing the domains of scalability solely within published journal articles should also be considered, as the content of journal publications are impacted by journal requirements. The ISAT identifies a variety of information sources that can be drawn upon for completing scalability assessments in addition to published literature, including any available evaluation reports, grey literature, practice-based information and expert option^(23)^. While it would be helpful for all studies to report on the domains of scalability, we recognise journal articles are not the sole source of information for policy makers and practitioners, who will likely use such evidence reported here, in addition to other sources of data (e.g. local data on workforce capacity, local policies) when making judgements to inform selection of interventions for scale. Additionally, while the current study provides an overview of which domains of scalability are reported within included journal articles, we did not systematically extract data relating to the content of each domain. Third, we employed a crude approach to categorising study stage of scale-up, based on study primary aims and outcomes, the delivery environment and delivery personnel. As the transition from efficacy to effectiveness exists on a continuum^(89)^, future reviews may benefit from employing a more comprehensive approach to classifying study stage of scale-up (e.g. PRagmatic Explanatory Continuum Indicator Summary-2)^(90)^, including assessment of a range of study design features (e.g. participant eligibility, intervention flexibility and analysis approach). Finally, although the issue of equity may be captured within the adaptation and reach domains, it is not explicitly assessed within the ISAT, and therefore within this review. Future scalability assessments should include consideration of the ability of an intervention to address health inequities (or ensure they do not contribute to the maintenance and/or exacerbation of health disparities at a minimum) as recommended by the WHO’s ExpandNET framework^(91)^.

Despite limitations, a strength of the review should be noted. While the psychometric properties of tools to assess the scalability of interventions are yet to be established^(21)^, we utilised the ISAT, one of the more comprehensive and methodologically sound tools for assessing scalability. The ISAT is considered to yield content validity as there was a well defined and rigorous process for developing tool content (including an explicit theoretical, conceptual and practical basis for the tool items and systematic item review by experts)^(22)^; and to only have minor methodological flaws, compared with the majority of scalability measures which have important methodological flaws^(21)^. Given increasing use of the ISAT^(24,25,83,86,92)^, the findings of this study yield relevant information for policy makers, practitioners, program managers and researchers and identifies gaps for researchers seeking to undertake research in the field.

### Conclusion

This review found that while a substantial number of RCTs have evaluated the impact of ECEC-based healthy eating interventions on child diet, the reporting of key scalability domains particularly cost/cost-effectiveness and sustainability remain scarce. At present, there is insufficient information for policy makers and practitioners to select ECEC-based public health nutrition interventions that are able to be delivered at scale, while maintaining meaningful effects on health outcomes. Reporting on all factors required for assessing scalability should be considered to support policymakers and practitioners selecting ECEC-based public health nutrition interventions for scale-up.
